# Enhancing interventions for prevention of mother-to-child- transmission of hepatitis B virus

**DOI:** 10.1016/j.jhepr.2023.100777

**Published:** 2023-04-24

**Authors:** Philippa C. Matthews, Ponsiano Ocama, Su Wang, Manal El-Sayed, Anna Turkova, Deborah Ford, Judith Torimiro, Ana Cristina Garcia Ferreira, Angélica Espinosa Miranda, Fernando Pio De La Hoz Restrepo, Emmanuel Seremba, Robinson Mbu, Calvin Q. Pan, Homie Razavi, Geoffrey Dusheiko, C. Wendy Spearman, Saeed Hamid

**Affiliations:** 1 The Francis Crick Institute, 1 Midland Road, London, NW1 1AT, UK; 2 Division of Infection and Immunity, University College London, Gower St, London WC1E 6BT, UK; 3 Department of Infection, University College London Hospitals, 235 Euston Rd, London NW1 2BU, UK; 4 Department of Medicine, Makerere University College of Health Sciences, Kampala, Uganda; 5 Cooperman Barnabas Medical Center, Florham Park, NJ, USA; 6 Hepatitis B Foundation, Doylestown, PA, USA; 7 Department of Paediatrics, Faculty of Medicine, Ain Shams University, Cairo, Egypt; 8 Medical Research Council Clinical Trials Unit, University College London, 90 High Holborn, London WC1V 6LJ, UK; 9 Chantal Biya International Reference Centre for Research on Prevention and Management of HIV/AIDS (CIRCB), Yaounde, Cameroon; 10 Faculty of Medicine and Biomedical Sciences, University of Yaounde, Yaounde, Cameroon; 11 Ministry of Health, Health Surveillance Department, Department of Chronic Diseases and Sexually Transmitted Infections, SRTVN Quadra 701, Lote D, PO700 Building, CEP: 70719-040, Brasília/DF, Brazil; 12 Universidad Nacional de Colombia, Bogotá, Colombia; 13 Division of Gastroenterology and Hepatology, NYU Langone Health, NYU Grossman School of Medicine, NY, USA; 14 Center for Disease Analysis Foundation, 1120 W South Boulder Rd Suite 102, Lafayette, CO 80026, USA; 15 Liver Unit, King’s College Hospital, Denmark Hill, London SE5 9RS, UK; 16 Division of Hepatology, Department of Medicine, Faculty of Health Sciences, University of Cape Town, South Africa; 17 Department of Medicine, Aga Khan University, Karachi, Pakistan

**Keywords:** HBV, hepatitis B virus, transmission, prevention, PMTCT, vertical transmission, HBIG, vaccination, tenofovir, birth dose, elimination

## Abstract

Prevention of mother-to-child transmission of hepatitis B virus (HBV) infection is a cornerstone of efforts to support progress towards elimination of viral hepatitis. Current guidelines recommend maternal screening, antiviral therapy during the third trimester of high-risk pregnancies, universal and timely HBV birth dose vaccination, and post-exposure prophylaxis with hepatitis B immunoglobulin for selected neonates. However, serological and molecular diagnostic testing, treatment and HBV vaccination are not consistently deployed, particularly in many high endemicity settings, and models predict that global targets for reduction in paediatric incidence will not be met by 2030. In this article, we briefly summarise the evidence for current practice and use this as a basis to discuss areas in which prevention of mother-to-child transmission can potentially be enhanced. By reducing health inequities, enhancing pragmatic use of resources, filling data gaps, developing advocacy and education, and seeking consistent investment from multilateral agencies, significant advances can be made to further reduce vertical transmission events, with wide health, societal and economic benefits.

**Key points**

• Prevention of mother-to-child transmission of HBV is a key component of global elimination strategies.
• Prevention of mother-to-child transmission of HBV using prophylactic antiviral therapy is only possible if HBV testing in pregnancy is accessible and affordable.
• Enhancing coverage of timely birth dose HBV vaccination is fundamental for prevention of mother-to-child transmission.
• Antiviral prophylaxis with tenofovir disoproxil fumarate is effective and low cost but is limited by poor access.
• Lowering the current viral load threshold and starting maternal prophylaxis before trimester 3 may bring benefits.
• Hepatitis B immunoglobulin for the infant is frequently not accessible due to the cost, cold-chain requirements, and difficulties in procurement.
• Evidence is needed to assess the impact of novel strategies (new tests and biomarkers, antiviral agents, neonatal antiviral prophylaxis).
• HBV prevention of mother-to-child transmission protects the neonate and may also optimise liver health for the mother, her partner, and other children.
• Successful prevention of mother-to-child transmission, with focus on populations at highest HBV risk, is cost effective and will redress health inequities.

## Background

Prevention of mother-to-child transmission (PMTCT) of hepatitis B virus (HBV) infection is a cornerstone of strategies for elimination of viral hepatitis. Specific international public health targets for HBV include 95% reduction in new infections in children,^1^ and lowering prevalence in 0-4 year olds from 0.9% in 2020 to 0.1% by 2030.^2^ PMTCT is crucial to tackle HBV infection and disease, as perinatal infection is associated with a >90% chance of chronic infection,^3^^,^^4^ with associated long-term risks of liver disease, including hepatocellular carcinoma (HCC) in young and middle-aged adults. Between one-third to one-half of all chronic HBV infections have traditionally been attributed to vertical transmission, but this proportion is increasing over time as a result of vaccination programmes that improve immunity in older age groups.^5^ Neonatal acquisition is now the major cause of chronic hepatitis B worldwide.

Thus, there is now an urgent need to review and revise practical, evidence-based approaches for HBV PMTCT. We set out to provide a summary of current evidence underpinning PMTCT recommendations, in order to highlight challenges, identify evidence gaps and promote potential advances in strategy. Elimination challenges differ between geographic regions, so lessons must be learned from existing experience and interventions tailored to find the best solutions for individual local contexts, while universally promoting efficacious, equitable and evidence-based interventions. Our approach aims to promote expert debate in areas that remain unresolved, inform further data collection and research, and provide advocacy for interventions, policy and funding to support best practice.

## Search strategy

To support our narrative review and inform opinions, we initially identified references through clinical guidelines and searches of PubMed, prioritising publications since 2015, but drawing on older primary literature where required for an evidence base. We used the search terms “Hepatitis B” and “HBV”, together with “mother to child transmission”, “mother to infant transmission”, “perinatal transmission”, “vertical transmission”, “elimination”, “pregnancy”, “birth”, “in utero”, “vaccination”. Material was also identified from the reference lists of included articles, conference abstracts and through searches of the authors’ own files. Only papers published in English were reviewed. The final reference list was generated on the basis of originality and relevance to the broad scope of this review.

## Risk reduction through PMTCT interventions

PMTCT recommendations are set out by the World Health Organization (WHO),^6^ and in regional HBV guidelines for Europe,^7^ the US,^8^ and Asia Pacific regions (Table 1).^9^ Interventions combine screening of pregnant women, antiviral prophylaxis with nucleos(t)ide analogues (NAs) for high-risk mothers from 28 weeks of gestation onwards, infant birth dose (BD) vaccination, and one or more doses of hepatitis B immunoglobulin (HBIG) administered to high-risk neonates, as a component of the WHO ‘triple elimination initiative’ to reduce the morbidity from HBV, HIV and syphilis.[10], [11], [12], [13] All infants should then subsequently receive a further three doses of HBV vaccine, combined into a multivalent formulation, as part of the routine WHO expanded programme for immunisation.

**Table 1:** **Summary of existing recommendations for use of antiviral agents and HBIG for HBV PMTCT**.

Guideline (date of publication)	Maternal prophylaxis/treatment recommendation	HBIG recommendation
South African guidelines (2013)^70^	• 3TC or TDF should be used in the third trimester in HBsAg-positive women with HBV DNA >1 x 10^6-7^ IU/ml. • Therapy can be discontinued 3 months post-delivery if only indicated for PMTCT	• HBIG (200 IU IM) to be administered within 12 h of delivery. • If the mother is HBeAg positive or has high HBV DNA, HBIG can be repeated at 1 month.
EASL (2017)^7^	• In pregnant women already on NA therapy, TDF should be continued (or other agents switched to TDF). • If HBV DNA >200,000 IU/ml (or HBsAg levels >4 log_10_ IU/ml), start TDF at week 24–28 of gestation and continue for up to 12 weeks after delivery. • No contraindication to breast-feeding.	• Give HBIG within 12 h of birth. • Specific indications/dose/regimen *etc.* not stated.
AASLD (2018)^8^	• Women who meet standard indications for HBV therapy should be treated. • If HBV DNA >200,000 IU/ml in the second trimester, consider treatment to prevent MTCT (preferably TDF). Recommendations are based on evidence for treating up to 12 weeks after delivery. • No contraindication to breast-feeding.	• HBIG and HBV vaccine should be administered to the newborn <12 h after delivery in high-risk cases including all low-birth-weight infants (but no specific definitions of high risk are specified). • HBIG advice refers to WHO and CDC recommendations.^147^^,^^148^
WHO (2020)∗^,^^6^	• For women with HBV DNA ≥5.3 log_10_ IU/ml (≥200,000 IU/ml), give tenofovir prophylaxis from the 28th week of pregnancy until at least birth.	• Give HBIG (if available) to infants of HBsAg-positive mothers, especially if HBeAg-positive or high HBV DNA.
Asia Pacific (2022)^9^	• LdT and TDF may be considered for mothers indicated for antiviral treatment during 1st-3rd trimester of pregnancy. TDF is preferred. • Antiviral prophylaxis is recommended if HBeAg positive or HBV DNA ≥200,000 IU/ml to start at 24-28 weeks of gestation. • NAs could be administered after discussion with the patient, even in patients with lower VL. • NAs could be stopped at birth or continued to 12 weeks post-partum.	• HBIG prophylaxis, in conjunction with HBV vaccination, may be of additional benefit for newborn infants whose mothers are HBsAg and HBeAg positive. • Variable access and recommendations recognised by country, recognising that where available, cost is often covered out-of-pocket.
UK ‘Green Book’ (updated 2022)^124^	• Treatment advice not provided by this source.	• Give HBIG for HBsAg+ women in any of the following categories; HBeAg positive; both HBeAg and anti-HBe negative; acute HBV infection during pregnancy; HBV DNA >1x10^6^ IU/ml in any antenatal sample; baby weighs <1,500 g at birth

CDC, Centers for Disease Control; HBIG, hepatitis B Immunoglobulin; HBeAg, hepatitis B e-antigen; HBsAg, hepatitis B surface antigen; HBV, hepatitis B virus; IM, intramuscular; LdT, telbivudine; MTCT, mother-to-child transmission; PMTCT, prevention of mother-to-child transmission; TDF, tenofovir disoproxil fumarate; WHO, World Health Organization.

HBIG guidelines are all based on post-partum IM administration of HBIG (0.5 ml), to be administered in combination with HepB-BD vaccine (though recommended at a different site).

∗ Many documents refer back to older WHO/CDC guidance from 1984,^121^ which pre-dates antiviral drugs and vaccination, to give HBIG preferably within 12 h of birth for HBeAg-positive mothers, with repeat HBIG at 3 months and 6 months to further reduce the probability of chronic infection.

Rigorous deployment of these interventions makes a substantial impact on vertical transmission rates (Table 2). Success has been exemplified in China, where – after an initial partnership with Gavi (the Vaccine Alliance) and subsequent government funding – a combination of improved antenatal HBsAg screening, provision of free BD vaccination, upscaling of the full vaccine schedule and deployment of village lay healthcare workers has reduced HBsAg seroprevalence in children <5 years from 9.7% in 1992 to <1% in 2013.^14^^,^^15^

**Table 2:** **Estimated impact of interventions on HBV MTCT; data summarised from peer-reviewed meta-analyses published in English over a 10-year period from 2013**.

Study location(s) (publication date)	Number of individuals represented	Prophylactic interventions for PMTCT	MTCT risk
**[I] Maternal antenatal prophylaxis**			

Worldwide; Brown *et al.*, 2016.^149^	3,622 pregnant women	Maternal NA prophylaxis	NA therapy reduced MTCT risk in infants at 6-12 months: - HBsAg seropositivity (RR 0.3, 95% CI 0.2–0.4) - HBV DNA seropositivity (RR 0.3, 95% CI 0.2–0.5)
Worldwide (majority in China); Li *et al.*, 2018.^150^	1,046 pregnant women	Maternal TDF prophylaxis	Compared to other NA or no NA prophylaxis, TDF reduced: - Infant HBsAg positivity rate (RR 0.25, 95% CI 0.16–0.38) - Infant HBeAg positivity rate (RR 0.26, 95% CI 0.14–0.48) - Infant HBV DNA positivity rate (RR 0.15, 95% CI 0.07–0.31) - Immunoprophylaxis failure rate (RR 0.31, 95% CI 0.13–0.73).
Worldwide; Khalighinejad *et al.*, 2019.^151^	2,667 pregnant women	Maternal 3 TC prophylaxis	Significant difference between the seropositive HBsAg infants between groups receiving prophylaxis *vs.* controls: RR 16.97, 95% CI 8.36-34.45.
Worldwide; Sali *et al.*, 2019.^152^	7,717 pregnant women; 7467 infants	Maternal NA prophylaxis (3 TC, LdT and TDF)	Overall, NA treatment reduced MTCT rate: - HBsAg positive infants at birth: OR 0.50, 95% CI 0.38–0.67 - HBV DNA positive infants at birth: OR 0.19, 95% 0.10–0.36 - Reduction in HBV positivity at 6 months: OR 0.15, 95% CI 0.11–0.19.
Worldwide; Song *et al.*, 2019.^68^	9,228 mother-infant pairs	Maternal NA prophylaxis (3 TC, LdT and TDF)	NAs reduced MTCT risk at birth: - HBsAg positive: RR 0.51, 95% CI 0.45–0.57 - HBV DNA positive: RR 0.22, 95% CI 0.18–0.26 No differences in efficacy of PMTCT between 3 TC, LdT and TDF. NAs more effective when administered from the second than from the third trimester based on HBV DNA (RR: 0.08 *vs.* 0.22)
Worldwide (majority in China); Wu *et al.*, 2020.^67^	6,738 pregnant women	Maternal NA prophylaxis (3 TC, LdT and TDF)	Compared to placebo: - 3 TC, LdT, TDF all reduced HBV MTCT: - In early pregnancy: RR 0.06; 95% CI 0.03–0.10 - In late pregnancy: RR 0.19; 95% CI 0.11–0.32 Timing of NA therapy: - Compared with initiation during trimester 3, MTCT was reduced when antiviral therapy was given earlier (RR 0.045, 95% CI 0.0053 to 0.20)
Worldwide; Funk *et al.*, 2021.^27^	TDF: 1,092 mothers/1,072 infants; 3 TC: 2,080 mothers/2,007 infants; LdT: 6,036 mothers/5,971 infants.	Maternal NA prophylaxis (3 TC, LdT and TDF)	Pooled ORs for RCTs: - 0.10 (95% CI 0.03-0.35) for TDF - 0.16 (95% CI 0.10-0.26) for 3 TC - 0.14 (0.09-0.21) for LdT

**[II] Caesarian section and/or avoidance of breastfeeding**			

Worldwide (focus on China); Yang *et al.*, 2017.^153^	9,906 pregnant women	Caesarian section (± HBIG and/or maternal NA prophylaxis)	MTCT rate - Overall: 6.76% - Caesarian group: 4.37% - Vaginal delivery group: 9.31% RR 0.51, 95% CI 0.44–0.60
Worldwide; Chen *et al.*, 2019.^154^	11,446 mother-child pairs	Caesarian section combined with immuno-prophylaxis	Average incidence of MTCT: - Caesarian group: 3.3% - Vaginal delivery group: 4.1% - OR 0.79, 95% CI 0.61–1.02 In the presence of immunoprophylaxis, no significant risk associated with vaginal delivery.
Worldwide; Pan *et al.*, 2020.^86^	3,429 participants (mode of delivery analysis); 2,443 participants (mode of feeding analysis)	Caesarian section and non- breastfeeding	In the absence of maternal antiviral prophylaxis, MTCT risk: - In Caesarean section group lower than vaginal delivery group: RR 0.58, 95% CI 0.46-0.74. - In the nonbreastfeeding group lower than in breastfeeding group: RR 0.74, 95% CI 0.56-0.98.
Worldwide, He *et al.*, 2022.^87^	11,144 women	Caesarian section	Caesarian *vs.* vaginal delivery at infant age >6 months OR 0.42, 95% CI 0.23–0.76

**[III] Infant immunoprophylaxis (passive HBIG, and/or active HBV vaccination)**			

China; Lin *et al.*, 2014.^155^	7,561 pregnant women	Vaccine and HBIG	MTCT risk despite immunoprophylaxis: - Overall 4.9% - In HBeAg-positive mothers: 9.7%
Worldwide; Jin *et al.*, 2014.^156^	4,274 antenatal prophylaxis; 1,061 postnatal prophylaxis assessed at birth 1,453 postnatal prophylaxis assessed at 7-12 months	Vaccine and HBIG	Antenatal prophylaxis: - Reduced MTCT (RR 0.36, 95% CI 0.28–0.45) at birth Postnatal prophylaxis: - Reduced MTCT: RR 0.66, 95% CI 0.52–0.84 at birth - Reduced MTCT: RR 0.54, 95% CI 0.42–0.69 at 7-12 months
Worldwide; Machaira *et al.*, 2015.^129^	3,426 pregnant women	Vaccine only, *vs.* vaccine + HBIG	MTCT risk in neonates who received vaccine only, compared with those who received vaccine + HBIG: - No difference in MTCT: OR 0.82, 95% CI 0.41–1.64.
China; Eke *et al.*, 2017.^157^	6,044 pregnant women	HBIG *vs.* no intervention	HBIG reduced MTCT compared with no intervention: - 6% MTCT with HBIG *vs.* 21% with no intervention; RR 0.30, CI 0.20–0.52
Worldwide; Chen *et al.*, 2020.^158^	2,440 pregnant women	Vaccine + HBIG	HBIG and vaccine group had a significant decrease in MTCT risk: - At birth: RR 0.2, 95% CI 0.18-0.40 - At age one year: RR 0.09, 95% CI 0.04–0.20

**[IV] Combined interventions**			

China; Xu *et al.*, 2014.^159^	2,033 pregnant women	HBIG, NA treatment, Caesarean section	HBIG *vs.* control: - Lower MTCT risk: RR 0.44, 95% CI 0.32–0.61 In NA prophylaxis group: - Lower MTCT risk: RR 0.06, 95% CI 0.01–0.24 In the vaginal delivery group: - Higher MTCT risk: RR 2.20, 95% CI 1.02–4.74
Worldwide; Chen *et al.*, 2017.^160^	2,706 infants	HBV vaccine from birth, HBIG + vaccine, antenatal NA prophylaxis	Reduced MTCT risk: - With HBV vaccine series RR 0.32; 95% CI 0.21–0.50). - With HBIG + vaccine *vs.* vaccine alone (RR, 0.37; 95% CI 0.20-0.67). - WIth antenatal NA prophylaxis *vs.* infant vaccine + HBIG alone if maternal HBV DNA >10^5^ IU/ml (RR 0.31; 95% CI 0.10–0.99).
Worldwide; Yao *et al.*, 2022.^60^	63,293 infants	Vaccination and maternal antiviral prophylaxis ± HBIG	Without prophylaxis, overall MTCT incidence: 31.3% (variable rates by region) Vaccination reduced MTCT risk: - In HBeAg-positive mothers from 82.9% to 15.9% - In HBeAg-negative mothers from 10.3% to 2.3% Maternal peripartum NA prophylaxis alongside infant immunoprophylaxis further decreased MTCT incidence to 0.3% (95% CI 0.1%-0.5%). Transmission risk stratified by HBV VL, with MTCT events documented at VL >4.29 log IU/ml

MTCT, mother-to-child transmission; RR, risk ratio; OR, odds ratio; RCTs, randomised-controlled trials; TDF, tenofovir disoproxil fumarate; 3TC, lamivudine; LdT, telbivudine; HBIG, hepatitis B immunoglobulin; VL, viral load.

Studies representing >1,000 individuals are listed, ordered according to the MTCT intervention, and then presented chronologically, starting with the oldest evidence.

However, the implementation of PMTCT measures lag far behind recommendations in many settings. There is poor awareness, lack of access to affordable tests for diagnosis and risk assessment, and limited availability of antiviral drugs, vaccines and HBIG, while the COVID-19 pandemic has disrupted existing immunisation programmes, widened health inequities, amplified vaccine hesitancy, and diverted attention and resource from HBV.[16], [17], [18], [19]

## Maternal testing and prophylactic antivirals: current practice

### Screening and risk stratification

Worldwide, most people living with HBV infection are not aware of their status;^20^ this also applies to pregnant women. Screening for HBV is recommended in all pregnancies,^6^ using a point of care test (POCT)^21^ or a laboratory ELISA to detect hepatitis B surface antigen (HBsAg) from venous blood or dried blood spots (DBS), but the advent of universal HBV vaccination has diminished the incentive for HBsAg testing of pregnant women in many regions of the world. HBsAg POCT has the advantage that follow-up samples can immediately be collected to stratify risk and inform management. This typically includes:

(i) HBV DNA viral load: A higher risk of vertical transmission is associated with maternal HBV DNA >200,000 (5.3 log_10_) IU/ml^22^^,^^23^ despite HBV vaccine series plus HBIG, and thus antenatal maternal NA prophylaxis is also recommended.^6^^,^^24^
(ii) HBV e-antigen (HBeAg): In situations where HBV DNA measurement cannot be undertaken, positive HBeAg is a proxy for viral load >200,000 IU/ml, with a reported sensitivity of 88% (95% CI 83.9–91.5) and specificity of 93% (95% CI 90.0–94.5).^24^

A decline in HBV DNA to <200,000 IU/ml by the time of delivery occurs in the majority of women who start NA prophylaxis at 28 weeks gestation.^25^ Data from randomised controlled trials (RCT) of tenofovir disoproxil fumarate (TDF), based on >1,300 pregnancies, reports MTCT rates of 0–6% in those receiving antiviral prophylaxis, compared to 2–30% in controls,^26^ while formal meta-analysis of RCT data generated pooled odds ratios of between 0.10 and 0.16 for different NA agents (Table 2).^27^

### Safety of antiviral therapy in pregnancy

The US Food and Drug Administration (FDA)^28^ reports no increased risk of congenital abnormalities associated with TDF, telbivudine (LdT), or emtricitabine (FTC) in pregnant women with HBV infection. A systematic review of the safety of antiviral HBV drugs in pregnancy collected data on neonatal outcomes (death, prematurity and congenital abnormalities, and also bone mineral density for TDF) and maternal outcomes (miscarriage/stillbirth, post-partum haemorrhage, antiviral resistance, and hepatitis flare), and found no evidence of increased risk for any of these adverse events.^29^

Tenofovir alafenamide (TAF) was approved for the treatment of HBV in adults and adolescents in 2016. TAF delivers the same active tenofovir metabolite as TDF, but at lower doses, thus reducing the incidence of side effects.^30^ Although TAF is thought to have a reduced renal and metabolic bone side-effect profile compared with TDF, it is potentially associated with weight gain, altered lipid profiles, and increased cardiovascular risk.^31^^,^^32^ However, in HBV PMTCT studies using TAF from gestational week 24 onwards, there were no significant side effects or toxicity, and no vertical transmission events.^33^^,^^34^ During breastfeeding, negligible concentrations of drug have been measured in breast milk, supporting its safety in ongoing treatment post-partum.^35^ The ALLIANCE trial reports an increased rate of favourable HBV endpoints (HBsAg loss, HBeAg seroconversion, alanine aminotransferase [ALT] normalisation) in previously treatment-naive HBV/HIV-coinfected individuals receiving a TAF regimen compared to a TDF regimen.^36^ However TAF has not yet been approved for HBV PMTCT and more data are needed. Furthermore, the high price of TAF means it is not yet a cost-effective choice even when healthcare is well-resourced.^37^

Experience accumulated through HIV ART also underpins important evidence for NA administration early in pregnancy, and provides information about the efficacy and safety of combination therapies (not well studied in HBV cohorts to date). A systematic review that collated outcomes on >19,000 pregnancies reported elevated risks of preterm delivery and low-birthweight infants when ART was started pre-conception *vs.* during pregnancy; however, this result may be confounded by other factors, and there was no evidence of other adverse outcomes, leading the authors to conclude that the overall benefits of ART outweigh the risks.^38^ A registry-based study in South Africa found no increased risk of congenital malformations in pregnancies exposed to first-line ART (including TDF/FTC) started prior to pregnancy *vs.* later,^39^ and in Botswana, this regimen was associated with better infant outcomes.^40^ Follow-up of >3,500 children in the US who were exposed to ART *in utero* did not identify any significant increase in adverse outcomes, up to the age of 13 years,^41^ corroborated by a more recent study of the impact of first-trimester TDF.^42^ Despite a possible association with a reduction in mean whole-body bone mineral content in the newborn,^41^ a double-blind RCT reported no evidence of a TDF effect on bone density in either mother or infant at 1 year of age,^43^ and other studies have also provided reassuring data.^44^^,^^45^

## Maternal interventions: challenges and data gaps

### Access to screening and stratification

Implementation of maternal risk assessment and antiviral therapy is currently impeded by inconsistency in routine antenatal HBsAg screening, and poor access to HBV DNA and/or HBeAg testing. Even when appropriate consumables and clinical/laboratory facilities are available, the costs of additional tests are often prohibitively high, either to the patient (where cost is out-of-pocket), to the state healthcare system (when there is government provision), and to private insurers (who may therefore not agree to cover costs).

HBeAg is typically cheaper than HBV DNA and more likely to be accessible, but it is not always a reliable proxy for HBV DNA quantification, particularly for certain viral genotypes.[46], [47], [48] Thus, among those testing HBeAg-negative, 12-16% of women typically have HBV DNA ≥5 log_10_ IU/ml and ∼2% have HBV DNA ≥7 log_10_ IU/ml,^24^^,^^27^^,^^29^ but the relationship between HBV DNA levels and HBeAg status varies between settings, at least partly dependent on the prevalent viral genotype (*e.g.* for genotype E, HBV DNA may be high in the absence of detectable HBeAg, while in other populations HBeAg positivity is associated with lower HBV DNA levels).^24^^,^^49^^,^^50^ Furthermore, HBeAg is subject to genomic, transcriptional, translational and post-translational changes. Other markers have been considered as potential substitutes for HBV DNA testing, including quantification of serum HBsAg (qHBsAg), or core-related antigen (HBcrAg) which correlates closely with HBeAg.^51^ However, these measures correlate imperfectly with HBV DNA (the picture is confounded by the fact that HBsAg is derived from both covalently closed circular DNA and replicating virus, but also from integrated viral genomes).^52^ Such tests are not yet widely available and more data are needed to determine their utility in PMTCT risk stratification.

In order to improve reliable and equitable risk stratification and reduce dependence on HBeAg, a rapid, affordable and POCT (or near-POCT) for HBV DNA is urgently needed. Infrastructure established for management of HIV infection has demonstrated that viral load testing can be made widely available. An inexpensive laboratory or POCT for HBV DNA could be added as a reflex follow-up to a positive HBsAg test, or could replace HBsAg screening, conferring the benefits of a single test that can be used for both diagnosis and risk stratification. A high sensitivity test is not required; a POCT for HBV DNA could be developed to provide a readout at the agreed threshold for treatment, with or without quantification over the range that informs use of antiviral prophylaxis and treatment. Long-term, broader access to testing for HBV DNA also allows for identification of cases of occult HBV infection (defined as cases in which HBsAg is negative, despite HBV viraemia). While occult HBV infection is not thought to be a significant contributor to MTCT, as HBV DNA concentrations are typically low, occasionally viraemia is at levels that are clinically significant and thus require treatment or prophylaxis.

In addition to HBV DNA testing, POCT for other biomarkers are also available or under development, with tests for detection and/or quantification or semi-quantification of HBeAg, HBsAg, HBcrAg and ALT. Careful validation of performance, accessibility, economic feasibility, and impact on care delivery is needed. A list of ‘prequalification’ tests has been produced by the World Hepatitis Alliance.^53^

### Treatment eligibility based on fibrosis scores

In individuals with evidence of significant liver fibrosis or cirrhosis associated with HBV infection, antiviral treatment should be started and continued irrespective of pregnancy (Table 1). However, identifying this group is challenging, as access to elastography is limited in many settings due to the high cost of hardware and software, alongside staff training and ongoing maintenance costs. Furthermore, elastography is not validated in pregnancy. Alternatively, estimation of fibrosis can be derived from laboratory parameters, such as APRI (aspartate aminotransferase-to-platelet ratio index) or FIB-4 (Fibrosis-4), but such scores have variable sensitivity and specificity, and further exploration is needed in diverse populations.^54^^,^^55^ A school of thought exists to suggest that infectious levels of virus observed in pregnancy may be an indication to continue treatment of the mother in her own right, irrespective of the level of serum aminotransferases or stage of fibrosis.

### HBV DNA thresholds for antiviral prophylaxis

Although a quantitative threshold has been determined for antenatal prophylaxis, in practice there is a progressive increase in MTCT risk with rising maternal HBV DNA levels, estimated as an odds ratio of 3.49 (95% CI 1.63–7.48) for each log10 increase in concentration, after adjustment for other factors.^22^ This observation, at least in part, accounts for the elevated risk of vertical transmission in younger mothers,^56^ in whom rates of HBV replication (and HBeAg) are higher. MTCT risk is also higher if HBV DNA is found in cord blood,^57^ and when acute maternal HBV infection occurs during pregnancy.^58^^,^^59^ Lowering thresholds for antiviral prophylaxis in pregnancy would reduce the risks of significant viraemia at term.

The current HBV DNA criterion for maternal antiviral prophylaxis is >200,000 (5.3 log_10_) IU/ml, but lower thresholds have been suggested, with ∼10,000 (4.0 log_10_) IU/ml as an indicative viral load at which infection may occur despite HBV vaccination if given alone without other PMTCT interventions.^60^ A lower threshold of viral load >2,000 (3.3 log_10_) has been proposed as a relaxed eligibility criterion for wider adult HBV treatment (irrespective of liver enzymes or fibrosis stage), and undetectable HBV DNA levels would mitigate any quantifiable transmission risk, based on the ‘undetectable = untransmissable’ (U=U) paradigm that is now widely applied to HIV.^61^ Particularly in settings where gaps in access to HepB-BD vaccine persist – consistent maternal screening and broadened eligibility criteria for prophylaxis could be of value to minimise the transmission risk. More evidence is needed to inform this agenda.

Offering prophylaxis to all pregnant women who test HBsAg positive has been explored. This approach removes the requirement for DNA or HBeAg testing to stratify risk, and provides opportunities to start treatment promptly, reducing the risk of delays or loss to follow-up, redressing health inequities, and preventing lifelong infection; economic analysis suggests this can be a cost-effective approach,^62^ though risk-benefit assessments in different populations are required.

### Starting and stopping antiviral prophylaxis

At present, NA prophylaxis is only recommended during the third trimester of pregnancy. However, the trajectory of decline in HBV viraemia on treatment is such that HBV DNA may not fall below 200,000 IU/ml by the time of delivery in a proportion of the population with high levels of viraemia (>30% in one study, although also impacted by incomplete adherence).^25^ Starting antiviral therapy earlier in pregnancy, potentially as soon as maternal HBsAg-positive status is confirmed, may therefore be advantageous in providing a longer period for virologic suppression (recognising that this can take weeks to months),^63^ providing better protection for babies born pre-term, and also possibly reducing the small risk of *in utero* HBV transmission events.^9^^,^^64^ Initiating TDF at gestational weeks 14-16 can suppress HBV DNA from >200,000 IU/ml to <20,000 IU/ml in 96% of cases,^65^ with no MTCT events and no increase in adverse events in mothers or infants.^66^ Meta-analyses suggest an advantage to starting lamivudine (3TC) at ≤28 weeks gestation compared to later in pregnancy,^29^ (although this agent is now less widely used for HBV due to resistance concerns). Other NA agents can be initiated in trimester one or two, without any adverse impact on safety (more details in Table 2).^67^^,^^68^ Clinical trials are in progress.^69^

Continuing maternal NAs post-partum provides a benefit for mothers in whom treatment is indicated by current guidelines, outside the context of PMTCT. In those who only required NA drugs for PMTCT, guidelines vary, with some recommending continuation for up to 12 weeks post-partum,^7^^,^^8^^,^^70^ while others suggest prophylaxis can be stopped at birth.^6^^,^^9^ However, in settings where HepB-BD delivery is not universal, stopping maternal medication before the infant receives at least one dose of HBV vaccination as part of multivalent infant vaccination poses a potential risk of horizontal transmission from mother to infant, and thus there is a rationale for continuing prophylaxis at least until this time point.

Recommendations for antiviral treatment of women before, during and after one or more pregnancies need to be informed by wider programmatic decisions about HBV treatment in all adults, with consideration of the potential individual- and population-level benefits of lowering the DNA threshold that determines eligibility.[71], [72], [73] In addition to reducing the risk of long-term liver disease in the individual, future strategies that endorse ongoing maternal antiviral therapy would also reduce MTCT risk for subsequent pregnancies, tackle the risk of horizontal transmission events within households, and abrogate the risk of severe liver flares associated with NA cessation. The optimal strategy will require pre-treatment HBV DNA quantitation, and the infrastructure to provide ongoing clinical monitoring and access to treatment for the mother; achieving this in practice is currently challenging in many settings but must be scaled up.

### Potential risks of NA prophylaxis

NA agents are generally safe and can be used in pregnancy, although more data would be advantageous to review outcomes of earlier and wider prophylaxis, and to provide better representation of diverse populations. If expanding NA prophylaxis, benefits must be weighed against the administration of medication to mothers with relatively low HBV DNA concentrations (where transmission risk should be mitigated by HepB-BD vaccination plus routine follow-up immunisation from 6 weeks of age). In settings in which neonatal and infant immunisation rates are poor, there is currently an argument for offering antiviral therapy to more pregnant women, while it also remains paramount to continue advocating for improved vaccine access.

There is a risk of hepatitis flares when treatment is stopped post-partum, with modest ALT elevations (*e.g.* with ALT rise to >2x upper limit of normal) in more than one-third of women,^74^^,^^75^ of whom some may seroconvert to anti-HBe or even lose HBsAg, and others may become eligible for long-term therapy. Interestingly, these flares may be less common in women who have received peripartum prophylaxis than in their untreated counterparts;^76^ reduced flares may be due to continuation of antivirals during the post-partum period. Most episodes of post-partum transaminitis are asymptomatic and resolve spontaneously;^77^^,^^78^ however, rare serious hepatitis resulting in morbidity and liver decompensation can occur.^79^ The risk is somewhat unpredictable, but is higher in younger and HBeAg-positive women.^78^^,^^80^ Access to a reliable, affordable, and consistent source of medication is therefore required.

Post-partum, it is impossible to gauge which mothers would benefit most from ongoing NA therapy unless HBV DNA quantification is undertaken at baseline. However, if overall guidelines for adult treatment are simplified, with wider treatment eligibility and access, this may help guide stratification and clinical management decisions during and after pregnancy. The cost of generic TDF (frequently <50 USD/per year in many countries) or, in the future generic TAF, should not be a barrier to treatment.[71], [72], [73]

### Single agent *vs.* combination prophylaxis

Although TDF is low cost and is included on the WHO list of essential medicines, there is poor access to tenofovir monotherapy in many settings where HBV is prevalent.^81^ Advocacy is urgently required to improve drug supply chains, with regional procurement tackled as a priority. Some clinical programmes have pragmatically capitalised on the availability of either TDF or TAF in combination antiviral preparations that are widely available for HIV treatment and pre-exposure prophylaxis, for example TDF/3TC. Furthermore, dual therapy could reduce HBV DNA more quickly and robustly than monotherapy, and emerging data show the potential benefits of combination therapy in chronic HBV infection,^82^ though there is no evidence base in pregnancy to date. Studies comparing single agent *vs.* dual therapy are warranted.

### Maternal HBV vaccination

In high endemicity settings, and in pregnant women at active risk of HBV exposure, it has been suggested that those testing negative could be offered HBV vaccination, which is safe in pregnancy and protects against acute infection (itself associated with a high risk of MTCT).^83^^,^^84^ However, such a programme consumes additional resources focused on the HBV-negative population, who should ideally be screened for anti-HBs and anti-HBc such that vaccination can be targeted at those who lack pre-existing immunity. In populations with a high prevalence of active HBV infection, the prevalence of prior exposure (anti-HBc) in the HBsAg-negative population is high, and routine catch-up vaccination for adults is not regarded as necessary or an appropriate use of resources.^85^ Approaches to maternal vaccination should therefore be reviewed based on risk assessment for individual women, alongside local epidemiology to inform population-wide strategies.

### Delivery route and breastfeeding

There may be a reduction in MTCT risk associated with Caesarian section *vs.* vaginal delivery, and in non-breastfeeding mothers^86^^,^^87^ (results of meta-analyses presented in Table 2). However, these interventions are not suggested for HBV PMTCT, as transmission risks can be adequately mitigated with other interventions, and there are other significant risks for mothers and babies associated with surgical delivery and with non-breastfeeding.

### HepB-BD vaccination: current evidence and interventions

HepB-BD has been incorporated into WHO guidance for all settings since 2009.^88^^,^^89^ At a cost of ∼0.25 USD per dose (UNICEF data for 2022),^90^ monovalent HepB-BD vaccination is deemed highly cost effective and has been described as the single most important intervention for HBV PMTCT,^91^ underpinning international targets for worldwide coverage of 90% by 2030.^1^ Modelling estimates suggest that >700,000 deaths in the 2020-2030 birth cohort could be prevented if this target were reached, with the greatest benefits in Africa.^16^ For complete protection, HepB-BD vaccination needs to be timely (soon after birth, and definitely within 24 h),^16^ and followed by two or three subsequent doses of vaccine, through the WHO Expanded Programme for Immunization (EPI). These subsequent doses are administered as a component of multivalent vaccines, typically starting at age 6 weeks, with a maximum recommended interval of 10 weeks between HepB-BD and the subsequent dose.^6^^,^^92^^,^^93^ Local capacity for vaccine manufacture is improving and a treaty for the African Medicines Agency was ratified in November 2021, with the aim of enhancing the continent’s regulatory and manufacturing independence.^94^

## HepB-BD vaccination: challenges and data gaps

### Logistical, financial and practical challenges

There are substantial ongoing challenges in delivery, consistency and equity of supply, maintenance of the cold chain, timely administration (particularly to babies born outside healthcare settings, in indigenous communities and in rural areas), and political acceptance of the need for HepB-BD vaccination.^95^ Despite global mandates, HepB-BD has not been consistently incorporated into regional or national guidance. In 2018, the coverage remained heterogeneous, globally estimated at 43%, and as low as 4-12% for the WHO Africa Region, where only 17/47 countries have formally adopted the strategy, compared to 75% in Latin America and >80% in the Western Pacific.^6^^,^[96], [97], [98] However, many African nations are making active progress towards introducing or enhancing HepB-BD vaccination, and there is a call for Gavi support to increase awareness of HBV and vaccination strategies, to train healthcare workers, and to enhance reach to infants born outside healthcare settings.^99^

Although monovalent HBV vaccine is low cost, total costs to healthcare systems include infrastructure and personnel, and the cost to the patient can be substantially higher, for example costing ∼10USD in Cameroon, which is met by the individual. Coverage can be enhanced by strengthening primary care infrastructure and increasing the proportion of deliveries that occur with trained birth attendants and access to healthcare settings. More data are needed regarding the optimum timing of vaccine administration, but there is recognition that early administration is likely to be most beneficial (for example within 1 h of birth),^100^ and that delay beyond 24 hours results in increasing risk.

There is increasing evidence for vaccine stability outside the cold chain for time-limited periods,^101^ allowing wider reach without concerns about reduced efficacy, and with demonstrable health-economic benefits.^102^ A ‘controlled-temperature-chain’ approach that allows vaccines to be held at ambient temperatures could replace the conventional stringent cold chain requirements.^103^ Relicensing the monovalent HBV vaccine to provide this flexibility will be key to enhancing coverage. Most vaccines cannot be frozen, which may also be a logistical challenge.

HepB-BD delivery falls outside the conventionally agreed remit of the EPI, and the lack of centralised ownership of the BD programme is a potential barrier to implementation. Scale up of political commitment, focused investment, infrastructure, resources and education is now required,^104^^,^^105^ which also depends on close integration with other aspects of maternal and infant care.^106^ However, data from epidemiological studies and modelling indicate that even high coverage of infant vaccination (including HepB-BD) is not sufficient to reach the goal of a HBsAg prevalence of <0.1% in children aged 5 by 2030,^5^^,^^107^ and this programme must be scaled up alongside other intervention strategies.

### Vaccine resistance

The impact of vaccine escape mutations has not been robustly studied in many settings, but tracking of the emergence and clinical significance of these polymorphisms will be important. Current sequencing data suggest a low prevalence of individual vaccine escape mutations, but there is a lack of viral genetic data to represent many populations.^108^ Unfortunately, HepB-BD vaccination is not infallible, as shown recently in Cameroon, where genetically engineered HBV A2 vaccines may not completely prevent MTCT from highly viraemic mothers infected with genotype E.^109^

### Stratification for HepB-BD vaccination

A risk-stratified approach to delivery of HepB-BD vaccination has been considered, *i.e.* targeting the intervention to infants born to HBsAg+ mothers, rather than to all infants. This focused strategy theoretically has a higher chance of attaining high coverage of infants at-risk, and success is being demonstrated in some settings (mainly in Europe) in which there is low HBV endemicity, high rates of antenatal screening, and consistent implementation of maternal prophylaxis and timely HepB-BD vaccination;^110^^,^^111^ use of this approach had been reported in 22 countries as of 2020.^112^ However, there are risks that not all infants would be sufficiently protected by this targeted approach in settings with higher endemicity and/or challenges in deployment of screening and prophylaxis, and it does not cover infants at risk of transmission events from other household or family members. Given the challenges associated with maternal stratification and targeted administration of timely HepB-BD vaccination, the WHO has continued to endorse a universal approach.^6^

## HBIG: current evidence and interventions

HBIG is a post-exposure prophylaxis strategy for infants born to high-risk mothers, ideally based on high HBV DNA and/or HBeAg-positive status when this stratification is available.^113^^,^^114^ A dose of 0.5 ml (at least 100 IU) is recommended for high-risk infants by many national and international guidelines (Table 1), most of which refer (directly or indirectly) to WHO/CDC guidance first published in 1984.^115^ HBIG requires storage at 2-8 °C (but must not be frozen),^116^ and has a limited shelf life (typically up to 1 year, but defined individually by suppliers). HBIG does not offer complete protection, particularly when the mother is HBeAg positive^57^ and/or has high HBV DNA in the third trimester,^23^^,^[117], [118], [119] but in combination with timely HepB-BD it reduces the rate of perinatal transmission from >90% to <10% in high-risk pregnancies.^7^^,^^120^ Follow-up HBIG doses may be advocated to further reduce the risk.^121^

## Neonatal post-exposure prophylaxis: challenges and data gaps

### Rationale for ongoing use of HBIG

HBIG has been deemed cost effective (in addition to universal vaccination) for some populations, but there are often issues with accessibility and feasibility, particularly in low-income settings.^9^^,^^122^^,^^123^ A mandate for HBIG administration can place a disproportionate burden on healthcare infrastructure and resources, and repeat HBIG doses are rarely practical due to the cost and difficulties in access. The theoretical possibility of transmission of prion disease has also contributed to scarcity of supply in some countries.^124^

HBIG recommendations have not been revised since the advent of other PMTCT interventions, but there has been a lack of studies formally assessing the efficacy of HBIG-free regimens.^27^ In the era of antiviral prophylaxis and HepB-BD vaccination, it is difficult to evaluate the impact of HBIG and signals for benefit frequently do not reach statistical significance.^125^^,^^126^ However, data are now emerging. A study in Singapore found no benefit of HBIG for the infants of HBeAg-negative mothers,^127^ while in Cambodia, HBIG offered no additional benefit if maternal TDF was started at least 4 weeks prior to delivery.^128^ A meta-analysis of nine studies of HBeAg-negative mothers in Asia and Europe found no significant difference in rates of MTCT between neonates who received HepB-BD vaccination with or without HBIG.^129^ In high-risk populations in Colombia, success in eliminating MTCT has been achieved without routine incorporation of HBIG.^95^ A multicentre study is underway to determine the impact of omitting HBIG from prophylactic strategies.^130^ To date, African populations have not been represented, and data are urgently needed as outcomes vary by setting and by viral genotype.

New guidelines must increasingly accommodate approaches that do not recommend routine HBIG use, focusing resources instead on strategies that are accessible, affordable, and sustainable. When HBIG is unavailable, maternal antiviral prophylaxis should be implemented as a priority, ideally supported by appropriate risk stratification, alongside timely HepB-BD vaccination.

### Neonatal prophylaxis with antiviral therapy

In HIV-exposed infants, post-exposure prophylaxis is given with ART for 4-6 weeks,^131^ setting a precedent for an approach that is widely deployed and accepted. To date, this strategy has not been explored for HBV, based on the alternative strategies of HepB-BD ± HBIG. However, recognising that timely delivery of vaccine and HBIG can be unreliable raises the question as to whether a defined course of antiviral prophylaxis could be a ‘safety net’ for selected high-risk HBV-exposed neonates.^109^ Where failure to administer HepB-BD vaccination is due to birth outside healthcare facilities, chances of successful administration of antiviral prophylaxis may be similarly low. Furthermore, the current lack of availability of paediatric formulations and access to treatment for infants is a potential barrier. Research is therefore needed to explore the risk/benefits, alongside feasibility, acceptability and health-economic implications of this approach in different settings. The REVERT-B study is currently exploring the role for neonatal 3TC prophylaxis and early maternal tenofovir.^132^

## Delivery of enhanced PMTCT interventions

### Enhanced approaches for HBV testing, antiviral prophylaxis and BD vaccination

**Fig. 1:**
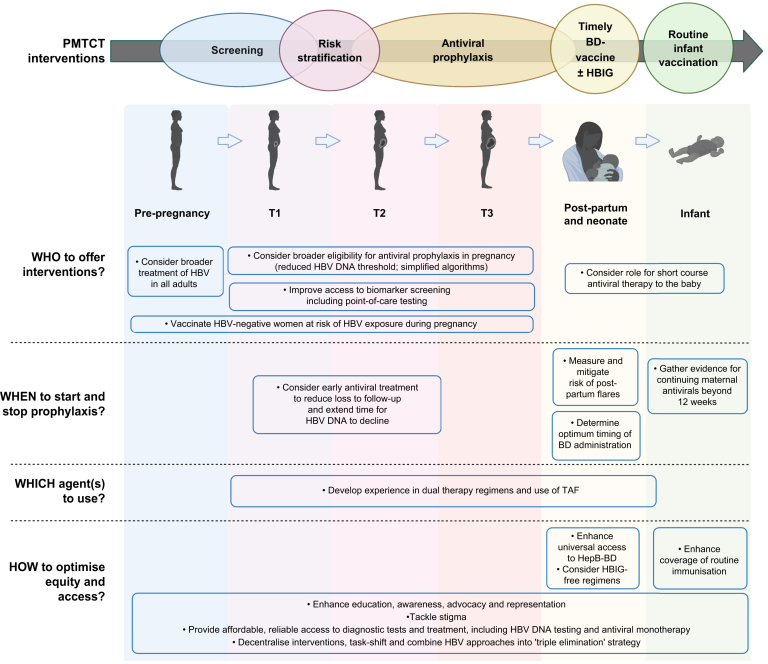
Areas for enhanced intervention in PMTCT of HBV infection. Graphic shows timeline before, during and after pregnancy, with existing interventions listed at the top, and areas where innovation is needed shown below. Triple elimination – combined interventions for prevention of HBV, HIV and syphilis. HBIG, hepatitis B immunoglobulin; HBV, hepatitis B virus; HepB-BD, hepatitis B birth dose immunisation; PMTCT, prevention of mother-to-child transmission; T1/2/3 - trimester 1/2/3 of pregnancy; TAF, tenofovir alafenamide.

An enhanced PMTCT strategy should ideally (i) improve screening through enhanced access to both HBsAg and reflex HBV DNA testing including POCT and quantification; (ii) support wider maternal access to antiviral agents, with evidence to determine the role of longer courses (pre- and post-partum), broadened eligibility criteria, dual therapy, and use of TAF; (iii) deliver universal timely HBV-BD vaccination (or, in low-endemicity settings, provide targeted BD vaccination if robust screening and linkage to care is deployed), and (iv) consider a role for infant post-exposure prophylaxis with NA agents as an alternative to HBIG (Fig. 1, Table 3). Specific interventions may need to be tailored according to different settings, based on local epidemiology of infection, resources and infrastructure. However, to make meaningful and sustainable advances, simplified approaches and pursuit of a combination of strategies is likely to be important. In settings where resources are limited, increasing awareness, political support based on the scientific evidence, training and investment are crucial. Continuing to assume that costs can be met by national or regional healthcare systems, or by communities, families or individuals, will lead to a failure of deployment and worsening of already harsh health inequities. Accepting the *status quo* is inadequate: there is an urgent need for strategies that deliver interventions that are practical, accessible, affordable and effective.

**Table 3:** **Recommendations for PMTCT of HBV infection and evidence gaps that need to be filled**.

Domain	Suggested interventions	Specific evidence gaps to be addressed^a^
**Diagnosis and risk stratification**	• **Invest in affordable and accessible diagnostics and risk-stratification tools.** ∗∗ • Simplify clinical algorithms such that assessment can be made by non-experts and in resource-limited settings.∗ • Caution regarding the risk of intra-uterine HBV transmission in pregnant mothers with high serum HBV DNA level (≥7 log10 IU/ml), which is increased in those who undergo invasive genetic testing procedures such as amniocentesis.	• Assess the availability of tools for diagnosis and risk-stratification (HBeAg, HBV DNA), with a focus on affordable POCT for high endemicity settings. • Develop and validate POCT for HBV DNA testing (quantitative or semi-quantitative). • Consider better biomarkers for risk assessment. • Evaluate risks of intra-uterine transmission, and provide education and information to healthcare workers and pregnant women about increased risks potentially associated with intra-uterine procedures.
**Antenatal vaccination**	• Consider HBV vaccination available prior to or during pregnancy for HBsAg-negative women at risk of exposure, to avoid acute infections during pregnancy.	• Assess feasibility, cost and acceptability of maternal vaccination during pregnancy in endemic settings/women with risk factors for exposure to acute infection.
**Prophylactic antenatal antiviral drugs**	• Consider offering antiviral prophylaxis to all antenatal women who are HBsAg-positive in settings in which further risk stratification is inaccessible.∗ • **Consider starting antiviral agent at the first point of contact in pregnancy in eligible women (especially in situations in which follow-up is challenging, and/or where the risks of transmission are known to be high).** ∗∗ • Recognise the use of combination antivirals for PMTCT (combining TDF with another HBV-active agent) on pragmatic grounds to extend access to therapy.∗ • Consider maintaining antiviral medication beyond the postpartum period, particularly in women who have not yet completed their family.∗	• Undertake formal evaluation (clinical/modelling) of the risks and benefits of antiviral agent started <28 weeks gestation, of dual antiviral therapy *vs.* monotherapy, and of TAF *vs.* TDF. • Gather evidence for benefits of treatment at VL <200,000 IU/ml in settings with/without routine HepB-BD and HBIG. • Enhance recommendations for best practice in high-risk situations, including (i) those who present late in pregnancy, and (ii) those with high HBV DNA who are HBeAg-negative. • Investigate acceptability of introducing antiviral agents earlier in pregnancy, and continuing for longer duration post-partum. • Evaluate risks of stopping NA post-partum (severe flare/fulminant hepatitis)
**Neonatal post exposure prophylaxis**	• **Invest in consistent and equitable supply of HepB-BD vaccine, and develop improved approaches to consistent implementation of timely administration.** ∗∗ • Follow up with vaccine doses (at 6, 10, 14 weeks ± 12-18 months) to achieve long term immunity.∗ • Advance progress on relicensing of monovalent HBV vaccine for use in a temperature controlled chain rather than cold chain.∗	• Consider whether neonatal antiviral prophylaxis is effective, acceptable and feasible as a ‘safety net’, e.g. in high-risk situations for infants of late-presenting mothers in settings with unreliable access to HepB-BD vaccine/HBIG. • Critically review the inclusion of HBIG in routine guidance for neonatal prophylaxis (as frequently unaffordable and inaccessible). • Evaluate whether earlier administration of HepB-BD (very soon after birth rather than up to 24 h) decreases MTCT risk.
**Education, advocacy and policy**	• Provide education so that populations and patients understand interventions, can advocate for diagnosis, treatment and vaccines, and are represented in policy-making.∗ • **Decentralise programmes, and combine services with those that tackle other infectious diseases and carcinogens, *e.g.* HIV PMTCT, BCG and OPV vaccination.** ∗∗ • **Develop ownership of interventions and resourcing from well established international vaccine programmes.** ∗∗	• Collect evidence for optimum approaches to education and engagement in different settings, involving diverse stakeholders (Community/health care workers/policy makers/funders); learn lessons from the implementation of HIV programmes. • Share knowledge and experience to amplify learning from case studies of successful PMTCT programmes.

HBIG, hepatitis B immunoglobulin; HepB-BD, hepatitis B birth dose vaccine; HBV, hepatitis B virus; HIV, human immunodeficiency virus; HPV, human papillomavirus; NA, nucleos(t)ide analogue; OPV, oral polio vaccine; PMTCT, prevention of mother-to-child transmission; POC, point of care; TAF, tenofovir alafenamide; TDF, tenofovir disoproxil fumarate; VL, viral load; XTC, 3TC (lamivudine) or FTC (emtricitabine).

a In all categories, there is a need for better data that represent the populations at highest risk, especially recognising evidence gaps for the WHO Africa region.

∗∗ Interventions marked with a double-asterisk and in bold are of the highest priority.

∗ Interventions marked with a single-asterisk are medium-ranked priorities.

### Partnerships, integration and decentralisation

Strong partnerships with industry are required to secure consistent access to diagnostics, treatments and vaccines, and affordable HBeAg and HBV DNA tests are undoubtedly a central requirement for providing safe, equitable, and evidence-based PMTCT interventions. There is a pressing need for programmatic approaches with HBV PMTCT interventions that are decentralised and integrated into other health interventions,^10^ including triple elimination, delivery of timely HepB-BD alongside other neonatal vaccines (neonatal BCG and oral polio), and provision of ongoing therapy for mothers and/or infants through established HIV infrastructure.^11^^,^^13^^,^^95^^,^^133^^,^^134^ Additional Gavi investment to support universal HepB-BD access (in addition to existing provision of multivalent vaccinations through the EPI schedule), and Global Fund ownership of screening pregnant women under the triple elimination initiative, would strengthen systems. Timely BD vaccination depends on training of healthcare workers who are present at the time of birth, requiring task-shifting to involve different personnel from those who deliver the routine paediatric immunisation schedule, and outreach into communities which specifically considers risks associated with home-births, rural communities, and season of birth.^135^^,^^136^ Financial incentives or subsidies could be provided to healthcare systems to boost coverage, while relaxation of strict cold chain requirements would support monovalent vaccine distribution in settings in which many births occur outside a healthcare setting.

### Equity and representation

PMTCT guidelines should account for differences between settings, and ‘conditional’ recommendations (as set out by the WHO) can be used to incorporate flexibility, such that approaches best serve the needs of specific populations. Enhanced data collection is required to determine baseline epidemiology and to track progress towards elimination targets (*e.g*. as described in Colombia).^95^^,^^137^ Current evidence typically overlooks high-risk populations, for example rural communities, migrant populations, and marginalised groups for whom there are barriers to healthcare access; there is an urgent need to extend the reach of clinical, public health and research programmes to close these equity gaps.^81^^,^^138^ The clinical and academic community, and patients and the public, should be uncompromising on demands for resources and intervention in order to drive high quality services with a clear focus on advocacy, universal access, and equity.

### Education and community partnerships

Stigma and discrimination associated with HBV infection are barriers to engagement, and PMTCT programmes for HBV have not been supported by appropriate education, awareness and community involvement.^139^^,^^140^ In some populations, there is no local word for HBV infection,^141^ highlighting a significant gap in awareness and knowledge. Practical implementation of PMTCT interventions is dependent on education of healthcare workers, and training birth attendants and local healthcare teams (*e.g.* community health extension workers).^136^^,^^142^^,^^143^ Informing the population and healthcare workers creates demand for interventions, and provides crucial community engagement and advocacy.^144^ There is typically a high level of acceptance of PMTCT interventions by women during pregnancy and post-partum,^145^^,^^146^ and this population can be strong advocates for themselves and their infants. PMTCT can be considered a family intervention, as identifying HBsAg-positive status in pregnancy also enables linkage to HBV care post-partum for the mother while providing the opportunity to screen (±vaccinate) partners, and other family members. These interventions function to break ongoing cycles of infection in families and communities.

## Conclusion

HBV PMTCT is a cornerstone of strategies to reduce the incidence and prevalence of HBV infection in children, lowering the population reservoir of virus (with an impact on reducing transmission), and reducing longer-term burden of disease, including cancer incidence. Tools are in place to abrogate PMTCT, but these have not been deployed adequately or consistently to date. There is now an urgent need to scale up existing interventions while tackling evidence gaps to optimise practice, reduce inequity and meet global targets for HBV elimination.

## Financial support

PCM is funded by the 10.13039/100010269Wellcome Trust (Grant ref 110110/Z/15/Z), UCLH NIHR Biomedical Research Centre, and receives core funding from the 10.13039/100010438Francis Crick Institute, London. AT and DF receive core funding from the UK Medical Research Council (grants MC_UU_00004/03).

## Authors’ contributions

Conceptualisation: PCM, CWS, SH. Resources: PCM, SW, AT, DF, CQP, CWS, SH. Writing – original draft: PCM, CWS. Writing – review & editing: all authors.

## Conflict of interest

CWS has received speaker fees from GILEAD Sciences and Abbott. CP received research funding and is a speaker for Gilead. PCM supervises a doctoral student with funding support from GSK. SH has received funding from Gilead for HCV Micro-elimination programs. HR has received research funding and speaker’s honoraria from Gilead Sciences, AbbVie and Pfizer and is a board member of the CDA foundation. SW has received research funding from Gilead Sciences, and honoraria from Prime Inc, and is on the Board of Directors for the Hepatitis B Foundation, World Hepatitis Alliance, and serves in the patient advisory group and HBV special interest group for the AASLD. FR has received consulting fees from Sanofi Pasteur. GD has received consulting fees and speaker fees from Gilead Sciences, has participated on Data Safety Monitoring Board/Advisory Board for janssen, Glaxo Smith Kline, Arbutus, Aligos, Vir and has roles in the National Medical Research Council Singapore and the World Health Organisation Pediatric Working Group on Viral Hepatitis.

Please refer to the accompanying ICMJE disclosure forms for further details.
